# Illuminating MXene quantum dots: from surface chemistry to white lasing *via* photoluminescence mechanisms and spectral engineering

**DOI:** 10.1039/d6ra01162d

**Published:** 2026-05-06

**Authors:** Usamah Sayed, Asmaa Edrees Fadhil, Subbulakshmi Ganesan, Subhashree Ray, Noor Mazin Basheer, Karthikeyan Jayabalan, Atreyi Pramanik, Apurav Gautam, Ahmad Mohebi

**Affiliations:** a Faculty of Allied Medical Sciences, Hourani Center for Applied Scientific Research, Al-Ahliyya Amman University Amman Jordan; b College of Pharmacy, Department of Pharmaceutical Sciences, AL-Turath University Baghdad Iraq; c Department of Chemistry and Biochemistry, School of Sciences, JAIN (Deemed to be University) Bangalore Karnataka India; d Department of Biochemistry, IMS and SUM Hospital, Siksha ‘O’ Anusandhan Bhubaneswar Odisha-751003 India; e Department of Medical Laboratory Technics, College of Health and Medical Technology, Alnoor University Mosul Iraq; f Department of Chemistry, Sathyabama Institute of Science and Technology Chennai Tamil Nadu India; g School of Applied and Life Sciences, Division of Research and Innovation, Uttaranchal University Dehradun Uttarakhand India; h Centre for Research Impact & Outcome, Chitkara University Institute of Engineering and Technology, Chitkara University Rajpura 140401 Punjab India; i Young Researchers and Elite Club, Tehran Branch, Islamic Azad University Tehran Iran a.mohebiacademic@gmail.com

## Abstract

MXene quantum dots (MQDs) have emerged as a distinctive class of zero-dimensional nanomaterials that combine strong quantum confinement with rich surface chemistry, enabling highly tunable photoluminescence (PL) properties. This review provides comprehensive mechanistic insight into the fundamental origins of PL in MQDs, emphasizing the interplay between core electronic structure, surface functional groups, edge states, and defect-mediated excited-state processes. Unlike conventional semiconductor quantum dots, MQD emission is governed by hybridized electronic states arising from transition-metal d orbitals coupled with surface terminations, heteroatom dopants, and hydrogen-bonded networks. We systematically analyze how surface chemistry, quantum confinement, and post-synthetic modifications regulate exciton formation, radiative and nonradiative recombination pathways, and excitation-dependent or excitation-independent emission behaviors. Advanced strategies for spectral engineering, including heteroatom doping, ligand passivation, defect control, and hybridization, are critically discussed in relation to multicolor emission, two-photon luminescence, and nonlinear optical responses. Special attention is given to recent breakthroughs in white emission and coherent white lasing enabled by MQDs, highlighting their potential in advanced photonic and optoelectronic applications. This review establishes a unified framework linking chemical design to excited-state engineering, offering guidance for the rational development of high-performance MXene-based luminescent nanomaterials.

## Abstract 2

MXene quantum dots reveal tunable photoluminescence arising from surface chemistry and quantum confinement. The review deciphers emission mechanisms and strategies enabling multicolor, nonlinear, and white lasing applications.

## Graphical abstract

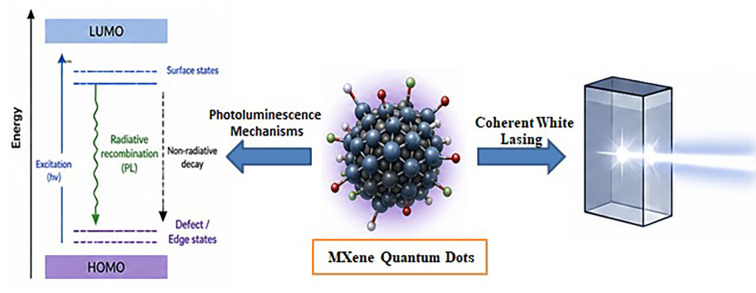

## 1 Introduction

Low-dimensional nanomaterials have revolutionized modern photonics and optoelectronics by enabling unprecedented control over light–matter interactions at the nanoscale.^1,2^ Among them, quantum dots (QDs) have attracted sustained attention due to their size-dependent electronic structures, tunable photoluminescence (PL), and broad applicability in lighting, sensing, bioimaging, and lasing technologies.^3–5^ While conventional semiconductor quantum dots rely primarily on bandgap modulation through quantum confinement, emerging low-dimensional materials have introduced new paradigms in which surface chemistry, defects, and hybridized electronic states play equally critical roles in governing optical behavior.^4–9^

MXenes, a rapidly expanding family of two-dimensional (2D) transition metal carbides and nitrides, have emerged as versatile materials with exceptional electronic conductivity, chemical tunability, and structural diversity.^10,11^ Derived from layered MAX phases, MXenes exhibit metallic or semi-metallic electronic structures combined with abundant surface terminations such as –O, –OH, and –F. While bulk and sheet-like MXenes are generally non-luminescent due to their metallic nature, the transformation of MXenes into zero-dimensional quantum dots fundamentally alters their electronic landscape.^12–14^ This dimensional reduction gives rise to MXene quantum dots (MQDs), which exhibit pronounced PL across the ultraviolet to near-infrared regions, opening new opportunities for nanoscale photonic applications.^15,16^

The emergence of PL in MQDs cannot be explained solely by classical quantum confinement models developed for semiconductor QDs. Instead, MQD emission originates from a complex interplay between quantum confinement, surface functionalization, edge chemistry, defect states, and hybridized electronic orbitals involving transition metal d states and surface p orbitals.^17–19^ The exceptionally high surface-to-volume ratio of MQDs amplifies the influence of surface chemistry, making chemical terminations, heteroatom doping, and post-synthetic modifications central to exciton formation and recombination dynamics. As a result, MQDs represent a unique class of luminescent nanomaterials in which chemical design and excited-state engineering are inseparably linked.^20,21^

In recent years, extensive experimental and theoretical studies have demonstrated that surface functional groups act as dominant luminescent centers in MQDs by introducing localized electronic states within the bandgap. Oxygen- and hydroxyl-terminated MQDs often exhibit enhanced PL through stabilized excitonic states, whereas excessive fluorine terminations may introduce nonradiative decay pathways.^22,23^ Edge states and structural defects, which are unavoidable in nanoscale MXenes, further contribute to excitation-dependent emission and multicolor photoluminescence. Moreover, heteroatom doping with elements such as nitrogen, sulfur, or phosphorus creates mid-gap states that enable excitation-independent emission and improved quantum yields. These findings collectively underscore that PL in MQDs is fundamentally governed by surface and defect-mediated processes rather than purely by size effects.^24,25^

Beyond fundamental photophysics, MQDs have demonstrated remarkable versatility in spectral engineering. Through rational control of size distribution, surface chemistry, and post-synthetic treatments, MQDs can be engineered to exhibit multicolor emission, two-photon luminescence, and nonlinear optical responses. Hydrogen-bonded surface networks, ligand passivation, and solvent-assisted modifications have been shown to suppress nonradiative recombination and stabilize excited states, enabling precise tuning of emission wavelength, lifetime, and photostability. These strategies have positioned MQDs as promising candidates for applications requiring controllable and robust light emission.^26,27^

One of the most significant recent breakthroughs in MQD research is the realization of white emission and coherent white lasing. Unlike traditional white-light systems that rely on phosphor conversion or multi-emitter integration, MQDs enable intrinsic white emission through the simultaneous activation of multiple luminescent centers within a single material platform. Ti_3_C_2_ MQDs have demonstrated excitation-dependent and two-photon white emission, while V_2_C MQDs have enabled coherent white lasing through nonlinear random scattering mechanisms.^28–30^ These advances highlight the potential of MQDs to function not only as passive fluorophores but also as active gain media in advanced photonic devices, including white lasers, ultracompact displays, and nonlinear optical systems.

Despite rapid progress, a unified mechanistic understanding links surface chemistry, electronic structure, and PL behavior in MQDs remains lacking. Existing studies are often fragmented, focusing on isolated aspects such as size effects, specific dopants, or individual applications. A comprehensive framework that connects chemical design principles to excited-state processes and spectral engineering strategies is urgently needed to guide rational material development and device integration.^31,32^

Despite the rapidly growing number of experimental reports on MXene quantum dots, the fundamental origin of their photoluminescence remains a subject of ongoing debate. Unlike conventional semiconductor quantum dots, MQDs do not exhibit a well-defined size-dependent bandgap governed solely by quantum confinement. Instead, their emission characteristics are strongly influenced by surface chemistry, edge states, defects, and hybridized electronic orbitals involving transition metal d states and surface p orbitals. This complexity has led to fragmented interpretations of MQD luminescence, with different studies attributing emission to confinement effects, surface functional groups, dopant-induced states, or defect-related transitions.^26–29^ The lack of a unified framework that systematically correlates chemical structure, electronic properties, and radiative mechanisms limits rational material design and hinders the optimization of MQDs for advanced photonic applications. Addressing this gap requires an integrated perspective that connects surface chemistry, electronic structure modulation, and exciton dynamics within MQDs, which remains insufficiently developed in current literature.

In this review, we systematically illuminate the fundamental PL mechanisms of MQDs, emphasizing the role of surface chemistry, hybridized electronic states, and defect-mediated exciton dynamics. We analyze how chemical and structural parameters govern radiative and nonradiative recombination pathways and discuss advanced strategies for spectral engineering, from multicolor emission to white lasing. By bridging fundamental insights with emerging photonic applications, this work provides a coherent perspective on the design of high-performance MXene-based luminescent nanomaterials and outlines future directions for their integration into next-generation optoelectronic and photonic technologies.

## 2 Chemical origin of luminescent centers in MXene quantum dots

Understanding the photoluminescence behavior of MXene quantum dots requires first identifying the fundamental chemical origins of their emissive centers. Since bulk MXenes are intrinsically metallic and do not exhibit significant photoluminescence, the emergence of light emission in their quantum-confined forms must arise from structural and chemical modifications occurring at the nanoscale. In particular, the high surface-to-volume ratio of MQDs introduces abundant surface terminations, edge sites, and structural defects that significantly alter their electronic landscape. Therefore, before discussing the detailed electronic processes governing emission, it is essential to establish the chemical factors responsible for generating luminescent centers. This section outlines how quantum confinement, surface functionalization, and defect-related states collectively contribute to the formation of radiative recombination pathways in MQDs.

MQDs represent a new frontier in two-dimensional material derivatives, where zero-dimensional confinement introduces unique optical, electronic, and chemical characteristics.^33,34^ While pristine MXenes exhibit metallic conductivity and layered structural motifs, their quantum dot forms display emergent luminescent behavior that is not present in bulk or sheet-like MXene counterparts. Understanding the chemical origin of luminescent centers in MQDs is essential for designing materials with tailored PL properties for applications ranging from optoelectronic devices to bioimaging.^35^

The luminescence in MQDs originates from a combination of quantum confinement effects, surface states, and chemical functionalization. Unlike classical semiconductor quantum dots, where the energy gap and emission properties are primarily dictated by size-dependent bandgap modulation, MQDs possess complex surface chemistry that introduces discrete energy levels capable of radiative recombination.^36,37^ The interplay between the intrinsic electronic structure of the transition metal carbide or nitride lattice and the chemical environment at the edges and surfaces is the primary determinant of PL characteristics.

**Fig. 1:**
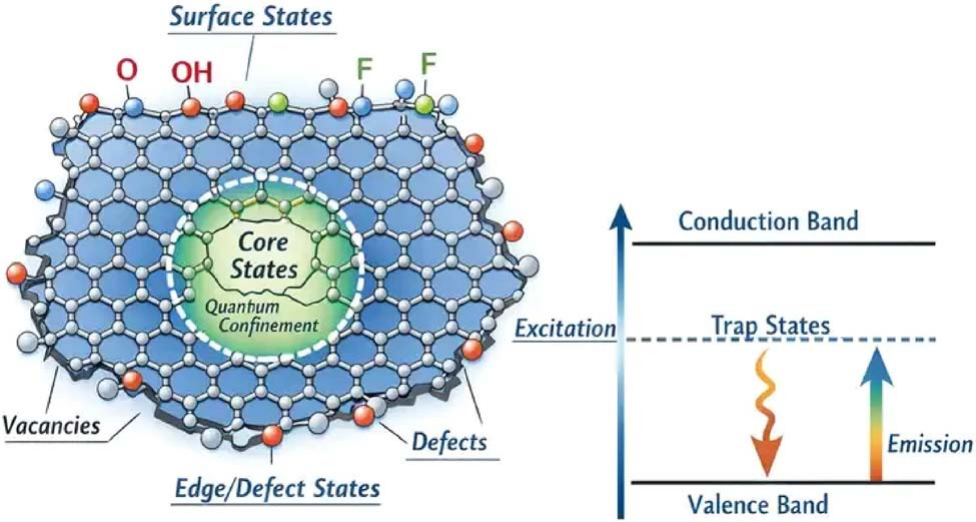
Schematic illustration of luminescent centers in MXene quantum dots showing contributions from quantum-confined core states, surface terminations, and defect-induced trap states governing photoluminescence emission.

A schematic representation of the chemical origin of luminescent centers in MQDs is illustrated in Fig. 1. The photoluminescence of MXene quantum dots originates from the combined contributions of quantum-confined core states, surface functional groups, and edge or defect sites. Due to the high surface-to-volume ratio of MQDs, surface terminations such as –O, –OH, and –F introduce localized electronic states within the bandgap. These states act as exciton trapping centers that facilitate radiative recombination. Additionally, structural defects and edge sites create trap levels that further contribute to excitation-dependent emission and tunable photoluminescence behavior.

### 2.1 Surface functional groups as luminescent centers

MXenes are inherently terminated with a variety of surface functional groups during synthesis, including hydroxyl (–OH), oxygen (=O), fluorine (–F), and other heteroatoms introduced through etching or post-synthesis treatment. In quantum dots, these surface groups dominate the electronic structure due to the high surface-to-volume ratio. Surface functional groups create localized electronic states within the bandgap, which serve as trap states for excitons. Radiative recombination from these surface states is a major source of observed PL.^38,39^

Hydroxyl and oxygen terminations typically introduce electron-withdrawing characteristics, modifying the local potential landscape and stabilizing excitonic states. Fluorine terminations, although often used to improve structural stability, can quench PL by introducing nonradiative pathways if not carefully controlled. The tunability of surface chemistry allows for modulation of emission wavelength, quantum yield, and even photostability. Moreover, heteroatom doping (such as nitrogen, sulfur, or phosphorus) can further create mid-gap states or modify the density of states near the Fermi level, enabling additional radiative transitions. These dopants often act as luminescent centers themselves or influence the local electronic structure to enhance exciton recombination probability.^40,41^ The chemical environment surrounding these dopants, including hydrogen bonding networks, solvent interactions, and adjacent functional groups, plays a pivotal role in determining the emission characteristics.

**Fig. 2:**
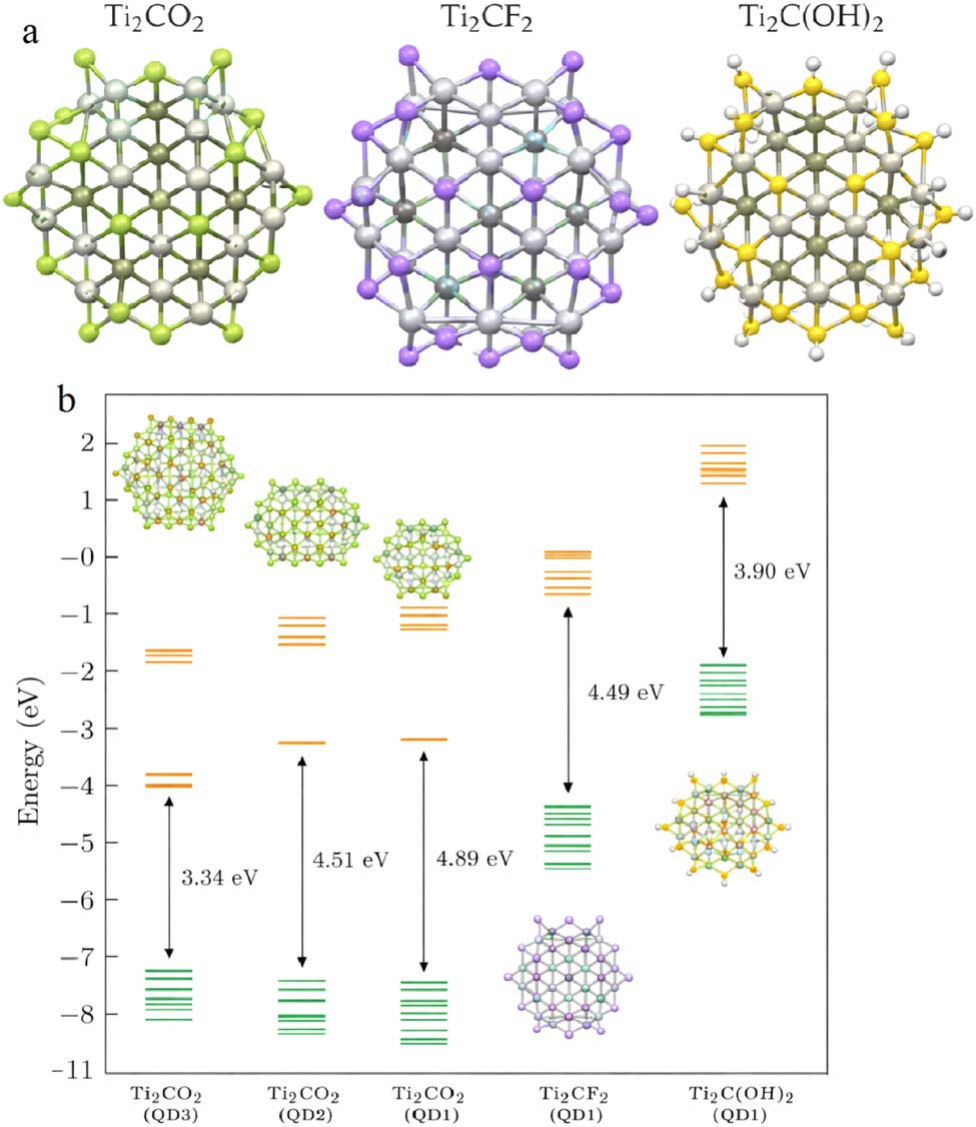
Optimized structures and energy levels of fully saturated Ti_2_CT_2_ MXQDs. (a) Atomic models of Ti_2_CO_2_, Ti_2_CF_2_, and Ti_2_C(OH)_2_ QDs illustrating preservation of the hexagonal core with different surface terminations (T = O, F, OH). (b) HOMO (green) and LUMO (orange) energy diagrams showing the influence of surface functional groups (Δ*g* = 4.89, 4.49, and 3.90 eV, respectively) and lateral size on the bandgap in Ti_2_CO_2_ QDs (QD1 to QD3), highlighting quantum confinement effects.

Panel (a) in Fig. 2 displays the optimized atomic structures of fully saturated Ti_2_CT_2_ MXene quantum dots (MXQDs) with different surface terminations: Ti_2_CO_2_ (left), Ti_2_CF_2_ (middle), and Ti_2_C(OH)_2_ (right). These models illustrate how oxygen (=O), fluorine (–F), and hydroxyl (–OH) functional groups bind to titanium atoms while preserving the hexagonal arrangement of the Ti_2_C core. Full saturation of the edges prevents structural distortions that would arise in partially or undersaturated QDs, ensuring structural stability critical for zero-dimensional systems where the high surface-to-volume ratio allows surface chemistry to dominate the electronic landscape. These functional groups introduce localized electronic states within the bandgap, acting as trap sites that promote radiative recombination and serve as the primary luminescent centers responsible for the observed photoluminescence in MXQDs.

### 2.2 Quantum confinement in zero-dimensional MXenes

Upon reducing the lateral dimensions of MXenes from tens of nanometers down to 1–5 nm, the electronic structure undergoes pronounced discretization due to quantum confinement.^42^ Confinement restricts the motion of electrons and holes in all three dimensions, leading to the formation of discrete energy levels rather than continuous bands.^43,44^ The energy separation between the highest occupied molecular orbital (HOMO) and lowest unoccupied molecular orbital (LUMO) increases as the size decreases, manifesting in a blue shift of the emission spectrum. This confinement-induced bandgap widening is further modulated by the lattice structure and elemental composition of the parent MXene, such as the transition metal (Ti, V, Nb, Mo) and the carbide or nitride composition.^45,46,86^

Quantum confinement alone, however, does not fully account for the high photoluminescence quantum yields (PLQY) observed in many MQDs. Unlike traditional semiconductor QDs, where exciton recombination occurs predominantly in the core, MQDs display significant contributions from surface and edge states, which act as additional luminescent centers. Therefore, understanding the chemical features of these surfaces is critical.^47,48^

Panel (b) in Fig. 2 shows the energy level diagram of occupied (green lines) and lowest unoccupied (orange lines) molecular orbitals for Ti_2_CT_2_ MXQDs, highlighting the combined effects of surface termination and lateral size on the HOMO–LUMO gap (Δ*g*). Oxygen termination yields the widest gap (4.89 eV), decreasing to 4.49 eV for fluorine and 3.90 eV for hydroxyl, while increasing QD size in the Ti_2_CO_2_ series progressively narrows the gap from 4.89 eV (QD1) to 3.34 eV (QD3). This size-dependent reduction demonstrates strong quantum confinement, which discretizes energy levels and induces a blue shift in emission for smaller dots. Together with surface states, confinement enables precise tuning of the bandgap and emission properties, explaining the significant contributions from edge and surface sites to high photoluminescence quantum yields beyond core-based recombination alone.

### 2.3 Edge chemistry and structural defects

In MQDs, edges and defects are inevitable due to mechanical exfoliation, chemical etching and hydrothermal or solvothermal synthesis methods. These edge sites often carry unsaturated metal atoms or partially coordinated carbon/nitrogen atoms. Such sites act as localized electronic states capable of trapping photoexcited electrons or holes. Radiative recombination from these edge states contributes significantly to the observed PL, often resulting in excitation-dependent emission spectra.^49,50^

Defects, such as vacancies, substitutional dopants, or lattice distortions, create additional discrete energy levels within the bandgap. Depending on their energy alignment, they can either serve as efficient radiative centers or nonradiative recombination centers. Controlling defect density through synthesis parameters is therefore critical for optimizing luminescence efficiency. Edge chemistry is also responsible for tunable emission color. Smaller MQDs with higher edge-to-bulk atom ratios tend to exhibit emission in the blue region due to stronger quantum confinement, whereas larger MQDs or those with functionalized edges may display green or red-shifted emissions, reflecting the influence of chemical and electronic heterogeneity.^51,52^

### 2.4 Interplay between core and surface states

Unlike classical semiconductor QDs where core states dominate PL, MQDs exhibit a strong interplay between core electronic structure and surface/edge states. The electronic coupling between the transition metal d-orbitals and p-orbitals of surface functional groups creates hybridized states, which can facilitate radiative transitions. This hybridization is highly sensitive to the chemical nature of the surface groups, MQD size, and structural distortions.^53,54^

Exciton localization at surface states can increase radiative lifetime and quantum yield, whereas delocalized excitons in the core may recombine nonradiatively. Therefore, the balance between core and surface contributions determines not only the emission wavelength but also photostability, lifetime, and quantum efficiency. Engineering this balance through chemical control is a central strategy for enhancing PL performance.^55,56^

**Fig. 3:**
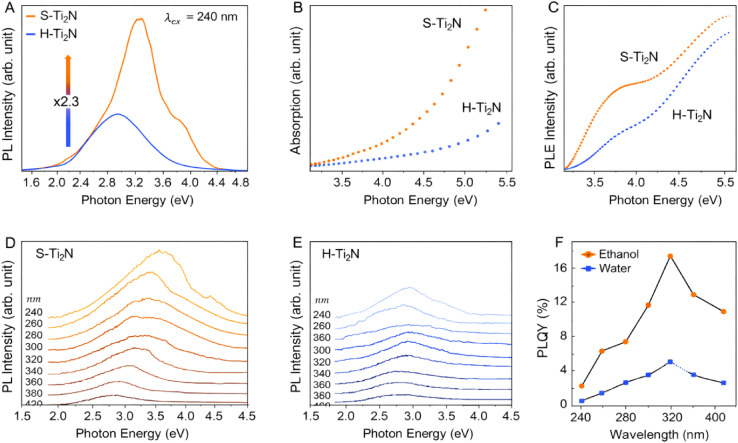
PL properties of S–Ti_2_N MQDs and H–Ti_2_N MQDs. (A) PL spectra under 240 nm excitation. (B) UV-vis absorption and (C) photoluminescence excitation (PLE) spectra. (D and E) Excitation-dependent PL spectra of S–Ti_2_N and H–Ti_2_N MQDs obtained with excitation wavelengths from 240 to 400 nm. (F) Quantum yields as a function of excitation wavelength, showing consistently enhanced luminescence efficiency for S–Ti_2_N MQDs. Adapted with permission from ref. 88 © 2025 Wiley.

Panel A in Fig. 3 presents the PL spectra of S–Ti_2_N MQDs and H–Ti_2_N MQDs excited at 240 nm. The S–Ti_2_N MQDs exhibit a markedly higher PL intensity, approximately 2.3 times that of the H–Ti_2_N MQDs, indicating a significantly enhanced radiative recombination efficiency. In addition, the emission peak of S–Ti_2_N MQDs is centered at a higher photon energy (∼3.4 eV) compared to H–Ti_2_N MQDs (∼3.0 eV), accompanied by a substantially narrower full width at half maximum. These features suggest a more uniform distribution of emissive states and reduced nonradiative losses in S–Ti_2_N MQDs, resulting in higher color purity and a smaller Stokes shift. Panels B and C show the UV-vis absorption and photoluminescence excitation (PLE) spectra of S–Ti_2_N MQDs and H–Ti_2_N MQDs, respectively. The absorption spectra reveal stronger absorption for S–Ti_2_N MQDs in the high-energy photon region, implying a higher density of optically active states. Consistently, the PLE spectra demonstrate that S–Ti_2_N MQDs maintain higher PL intensity over the entire excitation energy range from 3.2 to 5.5 eV. This behavior indicates that the enhanced luminescence of S–Ti_2_N MQDs is not limited to a narrow excitation window but originates from broadly accessible electronic states involved in both absorption and emission processes.

Panels D and E display excitation-energy-dependent PL spectra of S–Ti_2_N MQDs and H–Ti_2_N MQDs, obtained by varying the excitation wavelength from 240 to 400 nm. In both materials, the emission exhibits excitation-dependent behavior, reflecting the involvement of multiple emissive states. However, S–Ti_2_N MQDs consistently show stronger PL intensity across all excitation wavelengths, confirming the superior efficiency of radiative recombination. The relatively stable emission profile of S–Ti_2_N MQDs further suggests a more homogeneous surface-state landscape compared to H–Ti_2_N MQDs. Panel F summarizes the excitation-wavelength-dependent quantum yields (QYs) of S–Ti_2_N MQDs and H–Ti_2_N MQDs. S–Ti_2_N MQDs exhibit significantly higher QYs throughout the investigated wavelength range, reaching a maximum value of approximately 17.4% at an excitation wavelength of 320 nm. In contrast, H–Ti_2_N MQDs show considerably lower QYs under identical conditions. The pronounced enhancement in QY for S–Ti_2_N MQDs highlights the effectiveness of surface chemical modification in suppressing nonradiative pathways and promoting efficient photon emission. Taken together, the panel-resolved analysis demonstrates that sulfur incorporation in Ti_2_N MQDs profoundly alters the optical response by enhancing light absorption, stabilizing emissive states, and improving the balance between radiative and nonradiative recombination processes. The consistently higher PL intensity, narrower emission linewidth, and elevated quantum yield across multiple excitation conditions underscore the central role of surface-state engineering and core–surface electronic coupling in achieving high-performance MQD emitters through a simple solvothermal synthesis route.

### 2.5 Role of ligands, solvents, and post-synthetic modifications

Post-synthetic chemical treatments, including ligand attachment, solvent interactions, and thermal or chemical annealing, significantly influence the luminescent properties of MQDs. Ligands can passivate nonradiative defect sites, stabilize surface functional groups, and even participate in charge transfer with the MQD, thereby modifying emission properties. Solvent polarity, hydrogen-bonding capacity, and dielectric environment also affect exciton recombination dynamics.^57^ Annealing under controlled atmospheres can remove labile surface groups or restructure the MQD surface, creating a more uniform distribution of luminescent centers. Additionally, chemical hybridization with other low-dimensional materials (*e.g.*, graphene quantum dots, carbon nitride dots) can introduce energy transfer pathways, enhancing PL or enabling multicolor emission.^58,59^

### 2.6 Influence of transition metal composition and core electronic properties

The transition metal composition in MQDs critically determines their electronic structure and, consequently, their photoluminescent properties. Each transition metal contributes distinct d-orbital characteristics that influence the density of electronic states near the Fermi level, which governs how excitons behave within the quantum dot. Titanium-based MQDs typically exhibit higher bandgap energies, leading to blue-shifted emission, whereas vanadium, niobium, or molybdenum variants generally have narrower bandgaps, resulting in longer-wavelength emission. Mixed-metal or alloyed MQDs introduce further flexibility, as combining different metals creates new electronic environments and allows fine-tuning of the energy levels, effectively broadening the emission range.^60,61^

Beyond elemental composition, the intrinsic lattice structure and degree of crystallinity also influence electronic coupling between core states and surface orbitals. Highly ordered lattices enhance electronic delocalization, facilitating radiative recombination, while distorted or strained lattices can localize excitons, affecting emission lifetime and spectral width.^62^ Additionally, the interaction between the transition metal d-orbitals and surrounding surface atoms or functional groups establishes hybridized states that can act as additional luminescent centers. By understanding how each metal contributes to these core electronic interactions, researchers can strategically select or combine metals to design MQDs with targeted emission wavelengths, tailored spectral widths, and optimized PLQYs. This insight lays the groundwork for advanced chemical modifications that further modulate luminescent behavior while preserving the intrinsic contributions of the core electronic structure.^50,52^

### 2.7 Radiative mechanisms and chemical strategies for luminescence enhancement

PL in MQDs is governed not only by the core electronic structure but also by the interplay of surface, edge, and defect states that facilitate radiative recombination. The efficiency of light emission depends on the balance between radiative transitions and competing nonradiative decay channels. Surface functional groups such as hydroxyl, oxygen, and halogens introduce localized electronic states that trap excitons, enabling controlled emission, while excessive or improperly coordinated groups can create nonradiative pathways that quench luminescence. Heteroatom doping with elements like nitrogen, sulfur, or phosphorus introduces mid-gap states, opening additional recombination channels that enhance PL intensity. Structural defects, including vacancies or edge irregularities, similarly provide localized states that influence excitation-dependent emission spectra.^63,64^

Post-synthetic treatments—such as ligand exchange, solvent-assisted modification, or thermal annealing—further adjust the distribution and stability of luminescent centers, enabling control over emission wavelength, quantum yield, and photostability. Chemical hybridization with other low-dimensional materials, including graphene or carbon nitride dots, can introduce energy transfer pathways, expand the emission spectrum, and support multicolor photoluminescence. The combination of these strategies with the intrinsic electronic properties of the transition metal enables precise tuning of luminescence characteristics.^39,47^ This integration of chemical design and electronic structure engineering highlights the interdisciplinary nature of MQD research, where photophysics, materials chemistry, and surface science converge to produce quantum dots with predictable, customizable, and robust optical properties suitable for advanced applications in sensing, imaging, and optoelectronics.

### 2.8 Electronic properties and formation mechanisms of MQDs: comparison with semiconductor quantum dots

MXene quantum dots fundamentally differ from conventional semiconductor quantum dots in both their electronic structure and formation mechanisms. Semiconductor QDs, such as CdSe or PbS, derive their optical properties primarily from size-dependent quantum confinement within a covalent crystal lattice, resulting in a well-defined bandgap and excitonic recombination dominated by core states. In contrast, MQDs originate from metallic or semi-metallic parent MXenes, whose electronic structures are governed by transition metal d orbitals hybridized with carbon or nitrogen p states. Upon dimensional reduction to the zero-dimensional regime, lateral confinement discretizes these electronic states; however, the emergence of photoluminescence cannot be explained by confinement alone.^28,31^

The electronic properties of MQDs are strongly modulated by surface terminations (–O, –OH, –F), edge chemistry, and defects introduced during synthesis. Density functional theory calculations and spectroscopic studies consistently show that these surface functional groups introduce localized electronic states within the effective bandgap, acting as dominant radiative recombination centers. As a result, MQDs often exhibit excitation-dependent emission, broad spectral linewidths, and multiple emissive pathways—features that are uncommon in well-passivated semiconductor QDs.

The synthesis mechanisms of MQDs further distinguish them from semiconductor QDs. While semiconductor QDs are typically formed through bottom-up nucleation and growth processes that allow precise size control, MQDs are predominantly produced *via* top-down routes such as chemical etching, hydrothermal cutting, or solvothermal fragmentation of layered MXenes. These processes inherently generate abundant edge sites, defects, and heterogeneous surface terminations, which play a central role in defining the electronic and optical properties of MQDs. Consequently, chemical environment, etching chemistry, and post-synthetic modification exert greater influence on MQD luminescence than size alone.^35,39^

From an electronic perspective, MQDs occupy an intermediate regime between metallic nanoclusters and semiconductor QDs. Their photoluminescence arises from hybridized core–surface electronic states rather than purely band-edge excitons. This unique electronic architecture enables phenomena such as multicolor emission, two-photon luminescence, and intrinsic white emission within a single material system. Understanding these distinctions is essential for developing MQDs as chemically tunable light-emitting materials and highlights the necessity of treating MQDs as a distinct class of quantum-confined systems rather than as direct analogues of semiconductor quantum dots.

### 2.9 Classification and synthetic strategies of MXene quantum dots

MQDs represent a distinct subclass of low-dimensional nanomaterials derived from layered transition-metal carbides, nitrides, or carbonitrides. Due to their reduced dimensions and high surface-to-volume ratio, MQDs exhibit size-dependent electronic structures and rich surface chemistry, which strongly influence their optical and photoluminescent properties. A systematic classification of MQDs is therefore essential for understanding their structure–property relationships and guiding their synthesis.

From a structural perspective, MQDs can be broadly classified according to their parent MXene composition (*e.g.*, Ti_3_C_2_, Nb_2_C, Mo_2_C, and V_2_C), surface termination groups (–O, –OH, –F), and dimensional confinement. The specific transition metal layers largely determine the electronic density of states and d-orbital contributions near the Fermi level, while surface terminations modify the band structure, carrier localization, and optical transitions.^43–46^ In addition, MQDs can also be categorized based on their surface functionalization or heteroatom doping, such as nitrogen-, sulfur-, or phosphorus-modified MQDs, which are frequently introduced to tune emission wavelength, enhance quantum yield, and improve environmental stability.

The synthesis of MQDs generally follows two primary strategies: top-down and bottom-up approaches. The top-down strategy is the most widely employed route and involves the fragmentation or exfoliation of bulk MXene nanosheets into ultrasmall quantum dots. Common techniques include hydrothermal or solvothermal cutting, chemical oxidation, ultrasonic exfoliation, and electrochemical etching. During these processes, strong oxidation or mechanical forces break the layered MXene sheets into nanometer-scale domains while simultaneously introducing surface functional groups that play a crucial role in luminescence behavior. Hydrothermal treatment, in particular, enables controlled size reduction and defect engineering, which can enhance photoluminescence through the formation of emissive surface states.

In contrast, bottom-up synthesis relies on the nucleation and growth of MQDs from molecular or atomic precursors. Although less commonly used, this strategy offers advantages in terms of precise size control, uniformity, and tunable composition. Methods such as solution-phase chemical synthesis and templated growth have been explored to produce MQDs with controlled crystallinity and reduced structural defects.^24–27^ Bottom-up routes may also enable the incorporation of heteroatoms or hybrid structures during growth, providing additional opportunities to tailor electronic and optical properties.

Overall, the choice of synthesis strategy strongly influences the structural integrity, surface chemistry, and optical performance of MQDs. Understanding the relationship between classification, synthetic routes, and resulting electronic properties is therefore crucial for optimizing MQDs for applications in optoelectronics, sensing, and photonic technologies.

Table 1 provides a detailed overview of the primary chemical and structural parameters influencing PL in MQDs. Transition metals, lattice order, surface terminations, dopants, and defect sites collectively determine exciton dynamics and emission characteristics. Post-synthetic modifications and hybridization strategies allow fine-tuning of wavelength, quantum yield, and photostability. These combined factors highlight the interplay between core electronic structure and surface chemistry, enabling the rational design of high-performance MQDs for optoelectronic, bioimaging, and sensing applications.

**Table 1:** Key chemical and electronic factors governing PL in MQDs

Factor	Specifics	Photophysical impact	Typical range/notes
Transition metal composition	Ti, V, Nb, Mo, mixed-metal alloys	Modulates bandgap, emission wavelength, and exciton dynamics	Ti: 3.0–3.5 eV (blue emission), V/Nb/Mo: 2.0–2.8 eV (green-red shift)
Core lattice structure & crystallinity	Ordered *vs.* strained lattice	Affects electronic delocalization and exciton recombination	Highly crystalline → narrower emission, strained → broader emission
Surface functional groups	–OH, =O, –F, Cl	Create trap states, tune PL, can quench if excessive	Oxygen termination → blue-shift; –F → nonradiative losses
Heteroatom doping	N, S, P, halogens	Introduces mid-gap states, enables multi-color PL	Sulfur/N doping → excitation-independent emission
Edge sites	Unsaturated metal atoms, partially coordinated C/N	Localized states, affect excitation-dependent PL	Higher edge-to-bulk ratio → blue-shifted emission
Vacancies & defects	Carbon/nitrogen vacancies, lattice distortions	Can serve as radiative or nonradiative centers	Controlled defect density enhances QY
Post-synthetic modifications	Ligand attachment, solvent-assisted, annealing	Stabilizes excitons, tunes emission, enhances multicolor PL	Solvent polarity and ligand type critical for lifetime control
Hybridization & composites	Integration with GQDs, polymers	Creates energy transfer channels, expands emission spectrum	Enables stable multicolor emission and flexible device integration

## 3 Electronic structure and excited-state processes governing photoluminescence

Having established the chemical origins of luminescent centers in MQDs, the next step is to examine how these structural features influence the electronic structure and excited-state dynamics responsible for photoluminescence. The presence of quantum confinement, surface states, and defect-induced trap levels fundamentally modifies the density of electronic states and the distribution of charge carriers. These modifications determine how excitons are generated, localized, and recombine radiatively or non-radiatively. In contrast to classical semiconductor quantum dots, MQDs exhibit complex excited-state processes governed by the interplay between transition-metal d-orbitals, surface chemistry, and nanoscale confinement. This section therefore focuses on the electronic structure of MQDs and the fundamental excited-state mechanisms that ultimately govern their photoluminescent behavior.

### 3.1 Quantum confinement effects on electronic structure

The transition from bulk MXenes to quantum dots fundamentally alters the electronic landscape due to three-dimensional confinement. In MQDs, the spatial restriction of charge carriers leads to a discrete set of energy levels, modifying both conduction and valence band edges. Unlike classical semiconductors where the confinement is primarily size-dependent, in MQDs, the interplay between the transition metal d-orbitals and lattice composition determines the density of states near the Fermi level.^65^

The confinement-induced energy shift can be approximated by the effective mass model:^66,67^1
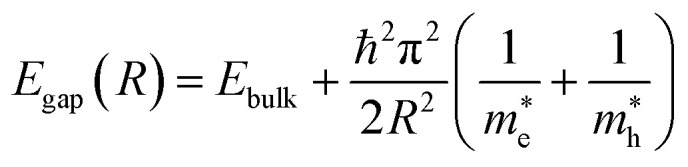
Where *R* represents the dot radius, and 
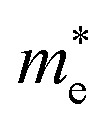
 and 
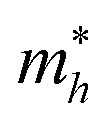
 are the effective electron and hole masses. This equation illustrates that smaller MQDs have significantly wider bandgaps, leading to blue-shifted emission. Importantly, this effect alone cannot explain the variety of observed PL phenomena, as additional excited-state interactions contribute to the emission profile.

Quantum confinement also enhances electron–hole overlap, which increases oscillator strengths and the likelihood of radiative recombination. Discrete electronic states in the DOS allow for selective absorption and emission transitions, which can be finely tuned through size control and transition metal selection.

### 3.2 Exciton formation and dynamics

Upon photon absorption, MQDs generate tightly bound excitons with significantly larger binding energies than their bulk counterparts. This is due to the combination of reduced dielectric screening and strong spatial confinement. Exciton binding energies in MQDs often exceed hundreds of meV, ensuring stability at room temperature. The overall exciton dynamics can be expressed as:^68,69^2
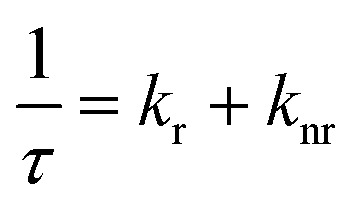
where *τ* is the exciton lifetime, *k*_r_ is the radiative decay rate, and *k*_nr_ is the nonradiative decay rate. In MQDs, exciton localization can occur both in the quantum dot core and at surface or defect states, but the localization is primarily determined by the electronic landscape shaped by the transition metal and quantum confinement.

Exciton dynamics are influenced by fine electronic interactions, including spin–orbit coupling and hybridization between metal d-orbitals and p-orbitals from neighboring atoms.^70^ These interactions determine whether recombination occurs radiatively, emitting a photon, or nonradiatively, transferring energy to lattice vibrations or phonons.

### 3.3 Density of states and electronic coupling

The electronic band structure of MQDs is inherently different from bulk MXenes. Transition metal d-orbitals dominate near the Fermi level, while carbon/nitrogen contributions create hybridized states that can participate in excited-state transitions. The discretized density of states (DOS) introduces multiple allowed transitions, each contributing to distinct emission wavelengths.^71^ The bandgap can be expressed as a function of quantum dot size using the effective mass approximation eqn (1).^72,73^

This formula shows that small variations in MQD size can significantly alter optical properties. In addition, electronic coupling between surface states and core states produces hybridized energy levels, which allow radiative recombination paths that are absent in bulk MXenes. The resulting PL spectrum may exhibit multiple peaks corresponding to different electronic transitions, enabling complex emission behaviors such as multi-color or excitation-dependent luminescence.

### 3.4 Radiative and nonradiative transitions

PL in MQDs arises from the competition between radiative and nonradiative processes. Radiative transitions occur primarily between discretized HOMO and LUMO states:^74,75^

3 *hν* = *E*_LUMO_ − *E*_HOMO_

where *hν* is the photon energy. Nonradiative decay can proceed *via* phonon-assisted relaxation, Auger recombination, or energy transfer to nearby quenching centers. The overall quantum yield is determined by:^76,77^4
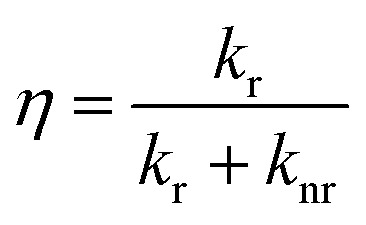


Engineering the balance between radiative and nonradiative pathways is key to achieving high PL efficiency. In MQDs, the presence of hybridized electronic states introduces multiple radiative pathways, allowing simultaneous emissions at different wavelengths, which is particularly useful for optoelectronic applications requiring tunable light sources. Nonradiative processes are further modulated by electron–phonon coupling. The strength of this coupling influences both the emission linewidth and exciton lifetime. Reduced electron–phonon interactions can lead to sharper emission peaks and longer-lived excitons, improving photostability and spectral purity.

### 3.5 Excitation-dependent and multi-color emission

Excitation-dependent emission is a hallmark of MQDs. Multiple discrete energy levels, arising from the combination of quantum confinement, metal d-orbital states, and surface hybridization, enable MQDs to emit at different wavelengths depending on the excitation energy. The relationship between emission wavelength and excitation energy can be approximated as:^78,79^5
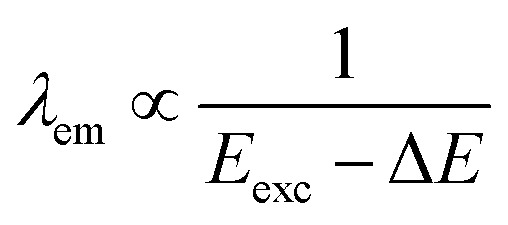
where *E*_exc_ is the excitation energy and Δ*E* accounts for internal relaxation within the MQD. This tunable emission allows MQDs to function as multi-color emitters in applications such as biological imaging, multiplexed sensing, and optoelectronic devices. Additionally, the excitation-dependent PL provides insight into the distribution of energy states, as transitions from different levels contribute to the overall emission spectrum. This property can be exploited to design MQDs with customized spectral profiles.

### 3.6 Energy transfer, coupling, and photophysical engineering

Excited-state processes in MQDs are also governed by energy transfer and electronic interactions with neighboring materials. Förster resonance energy transfer (FRET) between MQDs and acceptor species enables controlled quenching or enhancement of photoluminescence. The energy transfer rate can be expressed as:^80,81^6
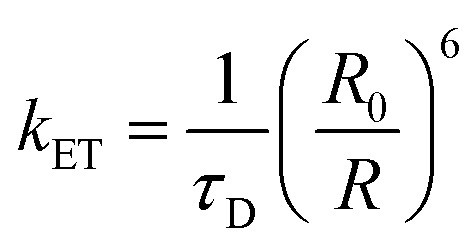
where *τ*_D_ is the donor lifetime, *R* is the donor–acceptor distance, and *R*_0_ is the Förster radius. Coupling to phonons or hybridized states introduces additional pathways that influence emission bandwidth, lifetime, and photostability. By tuning dielectric environments, inter-dot distances, or hybridization with other low-dimensional materials, researchers can engineer MQDs for desired PL characteristics. These strategies are particularly important for designing devices such as LEDs, lasers, and photodetectors, where precise control over emission wavelength, intensity, and lifetime is required.

Table 2 summarizes the principal physical processes controlling the photoluminescence of MQDs. Quantum confinement defines the energy levels and emission color, while defect- and surface-mediated exciton localization determines radiative or nonradiative recombination. The resulting PL depends on fine interactions between transition metal orbitals, surface chemistry, and dielectric environment. Understanding these interrelated processes is essential for engineering MQDs with predictable and tunable luminescent performance in optoelectronic devices.

**Table 2:** Key electronic and excited-state processes governing photoluminescence in MQDs

Mechanism/Process	Governing factor	Dominant effect on PL	Typical outcome
Quantum confinement	Dot size ®, effective mass	Controls bandgap widening	Blue-shifted emission
Exciton formation	Dielectric screening, confinement	Enhances exciton stability	Strong PL at room temperature
Density of states (DOS) modification	Transition metal d-orbitals, hybridization	Enables multi-peak emission	Excitation-dependent PL
Radiative transitions	HOMO–LUMO gap, oscillator strength	Promotes efficient emission	High quantum yield
Nonradiative decay	Phonon coupling, trap density	Induces quenching	Reduced emission intensity
Excitation-dependent emission	Energy level spacing, Δ*E* relaxation	Enables tunable spectra	Multi-color emission
Energy transfer (FRET)	Donor–acceptor coupling (*R*_0_, *R*)	Controls PL enhancement/quenching	Tunable intensity

## 4 Advanced applications and mechanistic insights of photoluminescent MQDs

The mechanistic understanding of luminescence developed in the previous sections provides a foundation for exploring how MQDs can be utilized in advanced photonic and optoelectronic applications. The interplay between quantum confinement, surface chemistry, and defect-induced electronic states enables highly tunable emission characteristics that can be engineered for specific technological purposes. By controlling synthesis conditions, surface functionalization, and hybrid material integration, researchers have demonstrated a wide range of photoluminescent behaviors in MQDs, including multicolor emission, nonlinear optical responses, and stable white-light generation. This section highlights representative applications and mechanistic insights that illustrate how the unique photophysical properties of MQDs translate into practical functionalities in emerging nanophotonic and optoelectronic systems.

### 4.1 Tunable two-photon white emission in Ti_3_C_2_ MQDs

Ti_3_C_2_ MQDs have emerged as promising nonlinear optical materials due to their intrinsic two-photon white emission, which opens new avenues for multiphoton imaging and ultracompact photonic devices.^82^ Synthesized through high-yield exfoliation followed by hydrothermal processing, these MQDs exhibit lateral dimensions of approximately 13 nm and a bilayer thickness. Their two-photon PL demonstrates exceptional stability under variable pressure conditions, allowing mechanical modulation from cool to warm white emission. The emission characteristics are highly sensitive to surface functionalization, structural uniformity, and defect density. Strong covalent and hydrogen bonding networks on the MQD surface stabilize the electronic states, thereby minimizing nonradiative relaxation and preserving the coherence of multicolor emission. This balance between structural integrity and electronic confinement is critical for designing white-light sources with predictable spectral output.

In addition, the incorporation of Ti_3_C_2_ MQDs into polymeric matrices, such as polydimethylsiloxane (PDMS), has significantly enhanced their processability and photophysical robustness.^82^ The polymeric hybridization not only maintains quantum efficiency but also provides mechanical flexibility, enabling the fabrication of stretchable and bendable white-emitting devices. Matrix embedding reduces aggregation, stabilizes excitonic states, and ensures uniform emission across the device, highlighting the importance of the interplay between chemical environment, nanoscale morphology, and optical performance. Such strategies emphasize that PL in MXene QDs is governed not only by intrinsic electronic structure but also by extrinsic chemical and mechanical modulation, creating a platform for engineering high-performance multiphoton emitters.

### 4.2 Hydrogen-bond mediated full-color emission in Ti_3_C_2_ MQDs

Full-color emission in Ti_3_C_2_ MQDs is achieved through strategic chemical doping and the formation of hydrogen-bonded surface networks, which modulate excitonic behavior and emission efficiency.^83^ Sulfur- and nitrogen-doped MQDs exhibit excitation-independent PL spanning the visible spectrum from blue to orange, a property essential for bioimaging and white-light-emitting diodes (WLEDs). The formation of robust hydrogen-bonded networks between bound water molecules and surface functional groups immobilizes surface bonds, reducing vibrational relaxation pathways and enhancing both quantum yield and emission lifetime. Spectroscopic analyses, complemented by computational studies, reveal that these networks increase structural rigidity at the nanoscale, which directly correlates with enhanced radiative recombination efficiency. By stabilizing excitonic states, chemical environment engineering allows precise tuning of emission wavelength without sacrificing photostability.

This approach illustrates the synergy between surface chemistry, quantum confinement, and photophysics in MXene QDs, providing a blueprint for rational design of full-color emitters.^83^ By modulating dopant types, density, and hydrogen-bond formation, it is possible to tailor optical properties for specific applications, including wavelength-specific imaging and tunable light sources. Furthermore, the independence of emission from excitation wavelength ensures reliable color output in device integration, a key factor in practical photonic and sensing technologies. These findings highlight how subtle chemical manipulation of MQD surfaces can produce macroscopic photophysical effects, bridging the gap between material chemistry and functional optoelectronic performance.

**Fig. 4:**
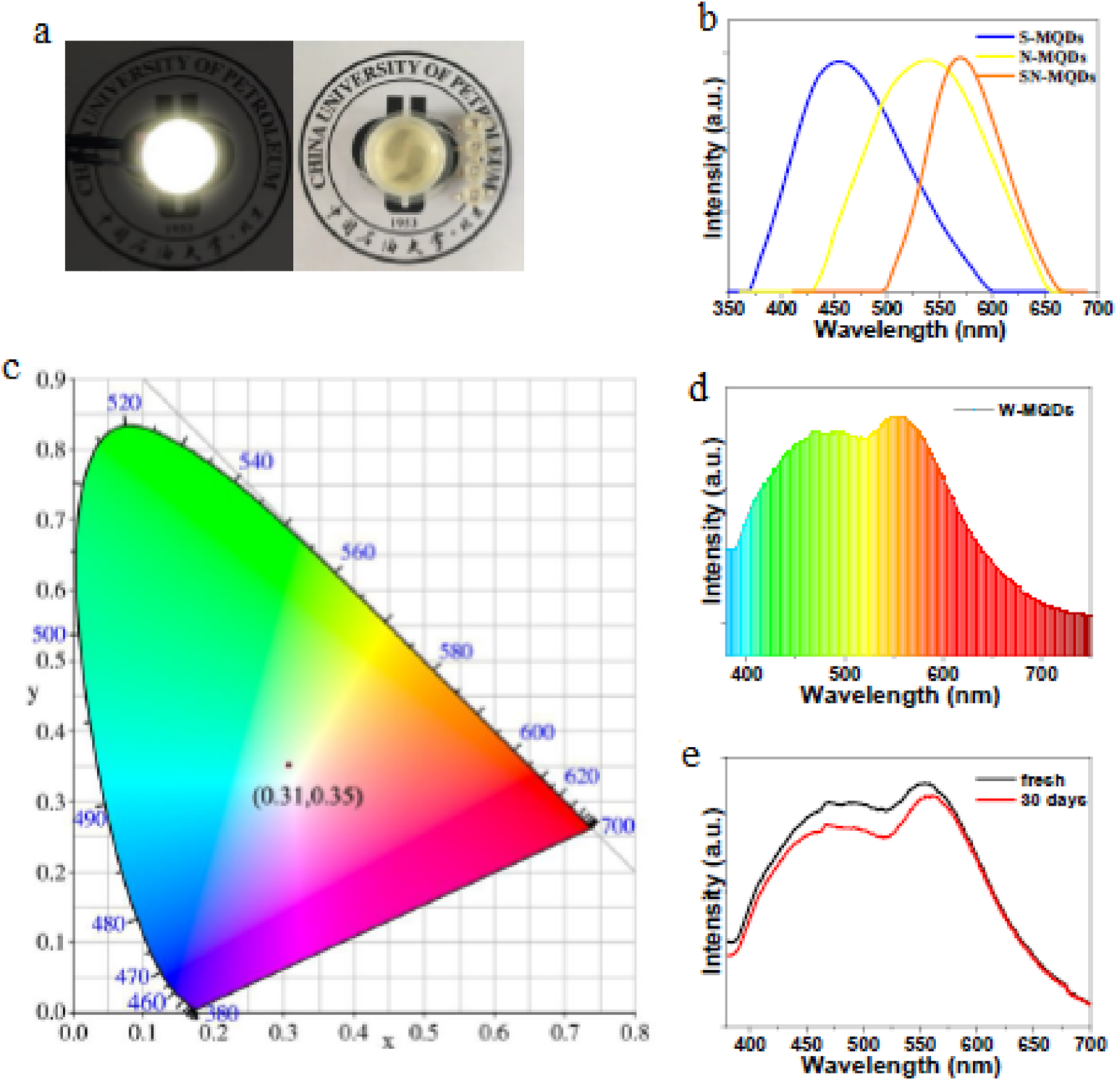
White-light emission from doped Ti_3_C_2_ MQDs. (a) Fluorescence images of W-MQDs/PVP composite under 365 nm UV illumination. (b) Normalized PL spectra of S-MQDs (blue), N-MQDs (green), and SN-MQDs (orange) under 360 nm excitation. (c) CIE 1931 chromaticity coordinates of W-MQDs at (0.31, 0.35). (d) Broad emission spectrum of W-MQDs. (e) PL spectra of fresh W-MQDs and after 30 days, demonstrating high photostability. Adapted with permission from ref. 83. © 2019 Elsevier B.V.

Fig. 4a shows fluorescence photographs of white-light-emitting MQDs (W-MQDs) incorporated into a polyvinylpyrrolidone (PVP) matrix, illuminated under 365 nm UV chips. The left image displays bright, uniform white emission from the composite coated on a commercial LED chip, while the right image reveals the same composite in a transparent PVP film. This visual demonstration highlights the successful integration of sulfur-doped (S-MQDs), nitrogen-doped (N-MQDs), and sulfur/nitrogen co-doped (SN-MQDs) Ti_3_C_2_ MQDs to achieve balanced full-color emission through homogeneous mixing, enabled by their excellent water solubility and compatibility with PVP. The uniform white light underscores the practical feasibility of these hydrogen-bond-mediated emitters for constructing stable white-light-emitting diodes (WLEDs).

Panel (b) presents the normalized PL spectra of the individual doped MQDs under 360 nm excitation, with emission peaks centered at 445 nm (blue, S-MQDs), 540 nm (green, N-MQDs), and 580 nm (orange, SN-MQDs). These broad, excitation-independent visible-range emissions arise from strategic chemical doping that introduces tailored surface states within the quantum-confined bandgap of Ti_3_C_2_ MQDs. The distinct peak positions reflect precise control over dopant-induced luminescent centers, allowing the combination of these primary colors to generate high-quality white light when blended, a critical feature for applications requiring tunable and reliable full-color output.

Panel (c) plots the Commission Internationale de l'Éclairage (CIE 1931) chromaticity coordinates of the W-MQDs/PVP composite under 360 nm excitation, positioned at (0.31, 0.35), close to the ideal white-light region. This near-central location confirms the effective balancing of blue, green, and orange contributions from the doped MQDs, facilitated by hydrogen-bonded networks that enhance structural rigidity and minimize non-radiative losses. The achievement of such coordinates demonstrates how surface chemistry engineering, through dopant incorporation and bound–water interactions, enables high-fidelity white emission suitable for advanced photonic devices.

Panels (d) and (e) illustrate the broad emission spectrum of the W-MQDs spanning 400–700 nm (d) and compare the PL spectra of fresh samples with those stored for 30 days (e). The negligible change in spectral shape and only minor intensity reduction over time highlight exceptional long-term photostability, attributed to robust hydrogen-bond networks that immobilize surface groups and suppress vibrational relaxation. This durability, combined with excitation-independent behavior and wide spectral coverage, positions these Ti_3_C_2_ MQDs as promising full-color emitters for stable, high-performance optoelectronic applications such as WLEDs and wavelength-tunable light sources.

### 4.3 Nonlinear random scattering and white lasing in V_2_C MQDs

V_2_C MQDs have recently demonstrated the capability for full-spectrum white lasing through nonlinear random scattering mechanisms, offering transformative potential for nanoscale photonics.^84^ By constructing a broadband scattering medium using excitation-power-dependent solvent bubbles, multiple emission bands—including blue, green, yellow, and red—are simultaneously amplified, resulting in coherent white lasing within a single gain medium. Surface passivation and defect density optimization are critical to balance gain across these wavelengths, preventing spectral broadening and nonuniform amplification. The localized nonlinear scattering induced by controlled MQD aggregation ensures efficient energy transfer between discrete photoluminescent centers, enabling coherent emission in ultracompact devices. These findings demonstrate that MXene QDs can serve as active photonic elements rather than passive fluorophores, with tunable lasing characteristics dependent on surface chemistry and nanoscale organization.

Moreover, careful engineering of the MQD environment—including optical density, size distribution, and defect control—allows precise control over emission coherence and spectral stability. The study highlights that the nonlinear optical properties of MQDs are intricately linked to their chemical and structural characteristics, emphasizing the importance of combining photophysical modeling with chemical surface design. Such systems have direct implications for full-color displays, multiband optical communication, and nanoscale sensors. By leveraging the interplay between quantum confinement, surface passivation, and excitation dynamics, V_2_C MQDs provide a versatile platform for high-performance white lasing at the nanoscale, representing a significant step forward in the application of MXene-based photonic materials.

### 4.4 Size and concentration dependent ultranarrow laser performance in Ti_2_C MQDs

The optical behavior of Ti_2_C MQDs is highly sensitive to both their lateral size and solution concentration, which directly influence ultranarrow photonics performance.^85^ Quantum dots in the range of 1–3 nm exhibit strong quantum confinement effects, resulting in discrete energy levels that enhance absorptivity and modulation depth. These properties are critical in achieving ultranarrow laser linewidths, as the optical gain is tightly linked to the density of states and exciton confinement within the MQD lattice. Higher concentrations increase the effective optical gain, yet introduce additional non-saturable losses that can broaden emission peaks if not carefully managed. Thus, the precise tuning of size distribution and dispersion concentration is essential for maintaining coherent lasing performance in loop-cavity setups, ensuring linewidths as narrow as 624.5 Hz with exceptionally high signal-to-noise ratios exceeding 77 dB.

Moreover, when these optimized MQD dispersions are integrated with erbium-doped fiber amplifiers, laser output can be boosted to approximately 60 mW, highlighting their potential in high-resolution photonics applications. This approach demonstrates that Ti_2_C MQDs can serve as active optical modulators rather than passive emitters, allowing dynamic control over light amplification at the nanoscale. The study further suggests that combining quantum confinement with precise surface functionalization enhances coherence and minimizes scattering losses, enabling applications in high-precision metrology, secure communication channels, and compact, ultranarrow-band photonic devices. Such advancements underline the importance of nanoscale control in tailoring optical properties for next-generation MXene-based photonics.

### 4.5 Surface termination effects on exciton confinement in Ti_2_C MQDs

Surface functional groups critically dictate the excitonic properties and optical absorption profiles of Ti_2_C MQDs, providing a versatile handle for tuning PL.^86^ Oxygen-, hydroxyl-, and fluorine-terminated MQDs exhibit distinctly different bandgap energies, with oxygen terminations producing the largest gaps and highest stability. Hydroxyl and fluorine terminations shift absorption toward visible and near-infrared regions, creating opportunities for tailored emission in diverse optoelectronic applications. The nanoscale size of these MQDs amplifies exciton binding energy, enhancing radiative recombination efficiency and producing blue-shifted emission. Surface engineering, therefore, becomes a powerful strategy to modulate exciton localization, recombination dynamics, and spectral output without compromising material stability.

In addition, combining quantum confinement effects with selective surface functionalization allows precise control over the first exciton energy and its delocalization across the quantum dot lattice. Computational modeling alongside experimental synthesis reveals that the interplay between intrinsic electronic structure and surface chemistry determines both emission wavelength and exciton lifetime. This tunability is critical for designing high-efficiency light-emitting devices, including LEDs, lasers, and bioimaging probes, where color purity, photostability, and quantum yield are all dependent on exciton management. Consequently, detailed understanding and manipulation of surface terminations in Ti_2_C MQDs establish a predictive framework for rational design of next-generation nanophotonic materials.

### 4.6 Low-temperature synthesized MQDs for controlled emission

**Fig. 5:**
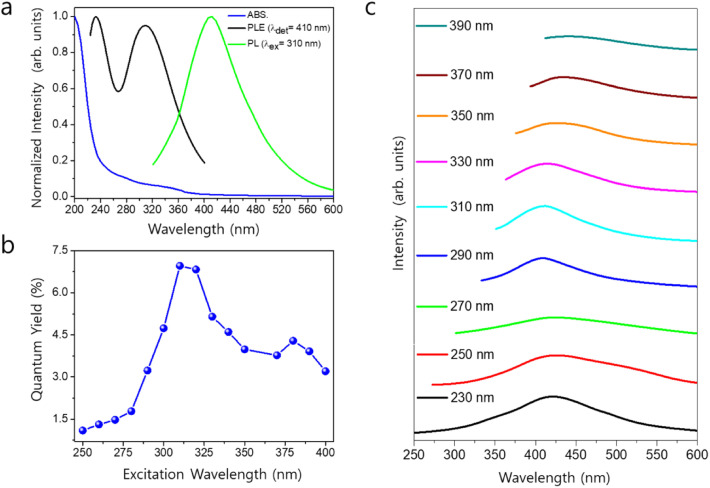
UV-vis absorption, PLE, and PL spectra (a), excitation-dependent PL quantum yield (b), and excitation-wavelength-dependent PL emission spectra (c) of low-temperature synthesized Ti_2_N MXene quantum dots, demonstrating controlled UV-driven emission behavior. Adapted with permission from ref. 87. 2022 MDPI.

Low-temperature synthesis of Ti_2_N MQDs provides an effective route to achieve uniform particle size (∼3.2 nm) and controlled PL behavior.^87^ Quantum yields of approximately 6.9% under 310 nm excitation confirm their potential for UV-range applications, where precise emission control is critical. Fig. 5a shows the UV-vis absorption, photoluminescence excitation (PLE), and PL spectra of the low-temperature synthesized Ti_2_N MQDs. The absorption spectrum exhibits two prominent absorption edges around 233 nm and 310 nm, which are well correlated with the corresponding peaks observed in the PLE spectrum. This agreement confirms that the observed PL originates from intrinsic electronic transitions within the MQDs rather than from surface defects or impurities. The strong absorption in the UV region highlights the suitability of these MQDs for UV-driven optoelectronic applications requiring controlled excitation pathways.

Fig. 5b presents the excitation-wavelength-dependent PLQY of the Ti_2_N MQDs. A maximum PLQY of approximately 6.9% is achieved under 310 nm excitation, which is consistent with the dominant absorption and PLE peak observed in Fig. 5a. This relatively high quantum yield, achieved through low-temperature synthesis, indicates efficient radiative recombination and minimal non-radiative losses. Such behavior demonstrates that precise control over emission efficiency can be achieved without high-temperature processing; reinforcing the advantages of low-temperature synthesis routes for functional MQDs. Fig. 5c illustrates the excitation-dependent PL spectra of the Ti_2_N MQDs over a wide UV excitation range (230–390 nm). The emission peak is centered around 420 nm, with a gradual red shift and reduced intensity observed as the excitation wavelength increases. This excitation-dependent PL behavior is attributed to quantum confinement effects and slight size dispersion among the MQDs, despite their overall uniform average size (∼3.2 nm). The ability to tune emission characteristics through excitation wavelength further confirms the controlled PL behavior of the low-temperature synthesized Ti_2_N MQDs, making them promising candidates for UV-based photonic and optoelectronic applications.

**Fig. 6:**
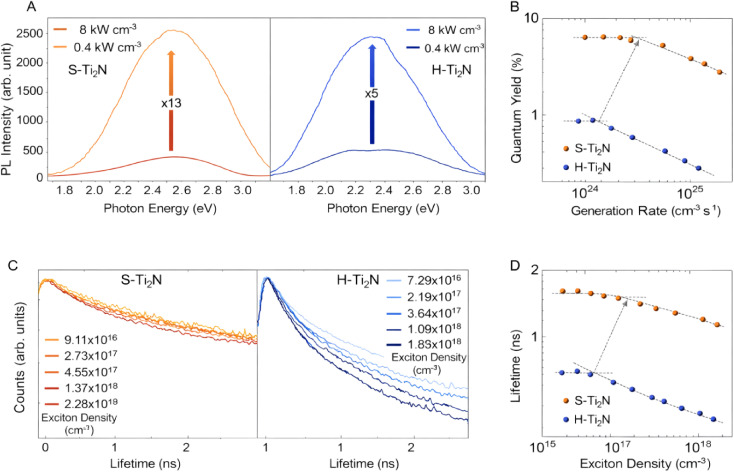
Excitation-dependent photoluminescence properties of low-temperature synthesized Ti_2_N MQD thin films highlighting superior Auger recombination resistance in solvothermal (S–Ti_2_N) MQDs. (A) PL spectra at low (0.4 kW cm^−3^) and high (8 kW cm^−3^) excitation intensities showing 13× and 5× intensity enhancement for S–Ti_2_N and H–Ti_2_N MQDs, respectively. (B) Quantum yield *versus* exciton generation rate, with delayed onset of QY decline in S–Ti_2_N MQDs. (C) Time-resolved PL decays at varying exciton densities. (D) PL lifetime *versus* exciton density, demonstrating slower lifetime reduction in fluorine-free S–Ti_2_N MQDs. Adapted with permission from ref. 88 2025 Wiley.

The solvothermal fabrication of fluorine-free Ti_2_N MQDs further improves UV emission efficiency, yielding peak emission at 370 nm with quantum yields up to 17.4% and superior resistance to Auger recombination.^88^ By eliminating fluorine-related surface defects, these MQDs maintain stable PL lifetimes even under high exciton densities, which is essential for high-intensity photonic and optoelectronic devices. Fig. 6 illustrates the excitation intensity-dependent PL properties of low-temperature synthesized Ti_2_N MQDs, comparing solvothermally prepared (S–Ti_2_N) and hydrothermally prepared (H–Ti_2_N) variants. Panel A displays the PL spectra for thin films of both types under 375 nm excitation at low (0.4 kW cm^−3^) and high (8 kW cm^−3^) intensities. For S–Ti_2_N MQDs, the PL intensity increases by a factor of 13, while H–Ti_2_N MQDs show only a 5-fold enhancement, despite a 20-fold increase in excitation intensity. This disparity highlights the reduced impact of Auger recombination in S–Ti_2_N MQDs, which are fluorine-free and synthesized *via* a solvothermal method that minimizes surface defects. Such behavior aligns with the controlled emission characteristics achieved through low-temperature synthesis, enabling uniform particle sizes (∼3.2 nm) and enhanced stability for UV-range applications as discussed in the context of precise PL modulation.

In Panel B, the QY is plotted against the exciton generation rate (*G*), revealing a gradual decline in QY for both MQD types as G increases, a common phenomenon in quantum-dimensional semiconductors due to non-radiative Auger processes. However, the onset of QY decline (marked by a gray arrow) occurs at a higher G for S–Ti_2_N MQDs, and the rate of decrease is slower compared to H–Ti_2_N MQDs. This superior resistance to Auger recombination in S–Ti_2_N MQDs stems from the elimination of fluorine-related defects during solvothermal synthesis, resulting in QYs up to 17.4% at peak emission around 370 nm. These findings underscore the effectiveness of low-temperature routes in optimizing surface chemistry, thereby improving emission efficiency and making these MQDs suitable for high-intensity photonic devices where maintaining high QY under stress is crucial.

Panel C presents TRPL spectra as a function of exciton density, showing decay curves for S–Ti_2_N and H–Ti_2_N MQD thin films. The H–Ti_2_N MQDs exhibit a pronounced shortening of PL lifetime with rising exciton density, indicative of intensified Auger recombination driven by charge imbalances and trap states. In contrast, S–Ti_2_N MQDs demonstrate minimal lifetime reduction across the same density range, reflecting the benefits of defect-minimized synthesis. This stability in PL decay supports the controlled emission profiles achievable through careful modulation of synthetic parameters like annealing temperatures and chemical environments, as emphasized in low-temperature approaches. Such attributes are vital for applications in flexible light-emitting devices, where operational reliability under varying excitation conditions is paramount.

Panel D quantifies the PL lifetime *versus* exciton density, with orange dots for S–Ti_2_N and blue dots for H–Ti_2_N MQDs. At lower densities (*e.g.*, ∼10^16^ cm^−3^), S–Ti_2_N MQDs maintain a lifetime of 1.65 ns, dropping only to 1.12 ns at higher densities (∼10^18^ cm^−3^), whereas H–Ti_2_N MQDs start at 0.49 ns and fall to 0.27 ns. The gray arrow indicates the density threshold for lifetime decline, which is higher for S–Ti_2_N, confirming slower Auger-induced degradation. This data illustrates how solvothermal, fluorine-free synthesis enhances resistance to non-radiative losses, aligning with the interdisciplinary strategies in materials science and photonics that tailor MQD properties for optimized quantum yields and durability in nanophotonic systems. The figure collectively demonstrates that low-temperature synthesized S–Ti_2_N MQDs outperform their H–Ti_2_N counterparts in mitigating Auger effects, leading to sustained high QYs and stable PL lifetimes under elevated excitation. These advantages arise from precise control over size distribution, surface states, and optical responses, facilitating tailored emission for UV photonics and high-density optoelectronics. By integrating chemical engineering with photonic design, such MQDs pave the way for next-generation technologies, where emission control and stress resistance are key to advancing functional capabilities.

These findings emphasize that careful control over synthetic parameters, including annealing temperatures, precursor preparation, and chemical environment, can systematically modulate MQD size distribution, surface chemistry, and optical properties. Such control allows for tailored emission profiles, optimized quantum yield, and improved stability under operational stress, enabling applications ranging from UV photonics to flexible light-emitting devices and high-density nanophotonic systems. The convergence of chemical engineering, materials science, and photonic design in low-temperature synthesized MQDs highlights the interdisciplinary strategies required to advance the functional capabilities of MQDs for next-generation optical technologies.

Table 2 presents a comparative analysis of MQDs highlighting their synthesis strategies, size, PL features, and potential applications. The data demonstrates how dimensional control, surface functionalization, and chemical doping influence quantum yields, emission spectra, and exciton confinement. Ti_3_C_2_ MQDs show both excitation-dependent and two-photon white emission, enabling flexible photonics and WLED applications, while V_2_C MQDs achieve coherent white lasing through nonlinear scattering, illustrating their potential in ultracompact full-spectrum lasers. Surface engineering *via* hydrogen-bond networks or fluorine-free synthesis further enhances PL stability and mitigates nonradiative pathways. Computational studies underscore the role of surface terminations and size on exciton binding energy and spectral tuning. Collectively, these findings indicate that precise chemical and structural control over MQDs is critical for advancing their performance in bioimaging, optoelectronics, and next-generation photonic devices Table 3.

**Table 3:** Comparative analysis of photoluminescent properties and applications of MQDs

Type of MQD	Synthesis method & conditions	Size/thickness	PL features & excitation	Quantum yield (QY)	Applications & remarks	Ref.
General MXene QDs	Review: hydrothermal, exfoliation, chemical etching	1–10 nm (typical)	Multi-color emission, tunable PL	High, variable	Overview of light-emitting MQDs; sensors, imaging, optoelectronics	89
Ti_3_C_2_ MQDs	Hydrothermal, mild conditions	3–5 nm/few layers	Excitation-dependent emission, strong quantum confinement	≈10%	Multicolor cellular imaging, Zn^2+^ sensing	90
Ti_3_C_2_ MQDs	High-yield exfoliation + hydrothermal	∼13 nm/2 layers	Two-photon white fluorescence, pressure-dependent shift	Not quantified	Hybrid PDMS devices; WLEDs; nonlinear optics	82
Ti_3_C_2_ MQDs (S-, N-doped)	Surface doping + hydrogen-bond networks	2–6 nm	Excitation-independent full-color PL (blue → orange), enhanced lifetime	Improved *vs.* dry MQDs	Bioimaging probes, WLEDs; hydrogen-bond networks stabilize PL	83
V_2_C MQDs	Random scattering system; excitation-power-dependent solvent bubbles	∼2–4 nm	Multicolor lasing; coherent white emission	Not quantified	White lasers on nanoscale; ultracompact full-spectrum emission	84
Ti_2_C MQDs	Size & concentration controlled dispersions	1–3 nm/few layers	Ultranarrow laser emission; strong absorption & modulation depth	Not quantified	Loop-cavity ultranarrow lasers; linewidth 624.5 Hz, SNR 77.63 dB; laser output ∼60 mW	85
Ti_2_C MQDs	TD-DFT modeling	1–2 nm	Exciton confinement; surface termination-dependent absorption; blue-shift with size reduction	Not quantified	Computational insights; oxygen termination largest gap; exciton binding energy up to 75%	86
Ti_2_N MQDs	Low-temperature two-step annealing + hydrothermal	∼3.2 nm	UV emission at 310 nm, narrow emission profile	QY ∼6.9%	UV-range photonics; low-temperature synthesis preserves surface states	87
Ti_2_N MQDs	Fluorine-free solvothermal	∼3 nm	UV emission at 370 nm, Auger-resistant	QY 17.4%	High-density exciton operation without PL decay; non-toxic, low-cost	88

### 4.7 MQDs in advanced optoelectronic devices

The unique electronic structure, tunable bandgap, and strong photoluminescence of MQDs have enabled their rapid integration into a variety of optoelectronic devices. One of the most widely explored applications is in light-emitting devices (LEDs). Due to their excitation-dependent emission and broad spectral tunability, MQDs can serve as efficient emissive materials for multicolor and white light generation. In particular, surface-engineered MQDs with controlled defect states have demonstrated enhanced radiative recombination efficiency, making them promising candidates for next-generation phosphor materials in solid-state lighting systems.

Another emerging application is in photodetectors. The strong optical absorption and fast carrier transport associated with the metallic or semi-metallic nature of MXene-derived structures enable MQDs to act as effective photoactive layers. When integrated with semiconductor substrates or hybrid heterostructures, MQDs can enhance photocarrier separation and extend spectral responsivity from the visible to the near-infrared region.^28–32^ The presence of surface functional groups also facilitates efficient charge transfer at interfaces, improving device sensitivity and response speed.

MQDs have also attracted attention in photovoltaic systems. Their tunable electronic states and strong light-harvesting capability allow them to function as sensitizers or interfacial modifiers in solar cells. In particular, MQDs incorporated into perovskite or organic solar cells have been reported to improve charge extraction efficiency and suppress nonradiative recombination at interfaces. Such effects can lead to enhanced power conversion efficiency and improved operational stability.

Furthermore, MQDs exhibit excellent performance in optical sensing platforms. Their photoluminescence is highly sensitive to environmental changes, including pH variations, metal ions, biomolecules, and chemical pollutants. Interaction with target analytes often modifies the surface states of MQDs, resulting in measurable changes in emission intensity or wavelength. This property has enabled the development of highly sensitive fluorescence-based sensors for environmental monitoring and biomedical diagnostics.^35–37^

Overall, the combination of tunable optical properties, rich surface chemistry, and solution processability positions MQDs as versatile materials for next-generation optoelectronic devices. Continued advances in synthetic control and surface engineering are expected to further enhance their device performance and broaden their application scope.

### 4.8 MQDs in advanced photonic and biomedical applications

Beyond conventional optoelectronic devices, MQDs have also demonstrated significant potential in advanced photonic and biomedical technologies. Their strong and tunable photoluminescence, combined with ultrafast carrier dynamics, makes them attractive candidates for photonic devices such as lasers and nonlinear optical systems. In particular, MQDs can serve as active gain media or optical modulators due to their broadband absorption and efficient radiative recombination. Experimental studies have suggested that MQD-based materials can support stimulated emission and potentially enable compact white-light or multicolor lasing systems when incorporated into suitable optical cavities.

MQDs also exhibit promising nonlinear optical properties, including saturable absorption and strong light–matter interaction. These characteristics allow them to function as optical switches or modulators in ultrafast photonic systems. For instance, MQD-based saturable absorbers have been explored for generating pulsed laser outputs in fiber laser configurations. The ability to engineer surface states and electronic structures further enables the tuning of their nonlinear optical response.^19–24^

In addition to photonic technologies, MQDs have gained increasing attention in biomedical applications. Their nanoscale size, large surface area, and tunable fluorescence make them promising probes for bioimaging and biosensing. Compared with conventional semiconductor quantum dots, MQDs can exhibit improved aqueous dispersibility and surface functionalization capability, which facilitates conjugation with biomolecules such as antibodies, peptides, or nucleic acids. As a result, MQDs can be engineered as fluorescent probes for cellular imaging, targeted detection of biomolecules, and monitoring of biological processes.

Moreover, the photothermal and photodynamic properties of certain MXene-based nanostructures suggest potential therapeutic applications. MQDs capable of converting light into heat or reactive oxygen species may serve as platforms for photothermal therapy or combined diagnostic-therapeutic (theranostic) systems. These multifunctional capabilities highlight the versatility of MQDs in emerging biomedical technologies.^31–34^ Taken together, the integration of MQDs into photonic and biomedical platforms demonstrates their potential beyond traditional optoelectronic systems. Continued investigation into their optical dynamics, surface chemistry, and biocompatibility will be essential for translating these materials into practical photonic devices and biomedical tools.

## 5 Challenges and limitations of MXene quantum dots

After discussing the chemical origins, electronic mechanisms, and emerging applications of photoluminescent MQDs, it is important to synthesize these insights to identify key challenges and future research directions. Although significant progress has been achieved in understanding MQD photophysics, several fundamental questions remain regarding the precise role of surface chemistry, defect engineering, and excitonic dynamics in governing emission efficiency and spectral tunability. Furthermore, scalable synthesis strategies and improved structural control are essential for translating laboratory-scale discoveries into practical devices. This final section therefore summarizes the major insights presented throughout the review and outlines promising research avenues that may enable the rational design of next-generation MXene quantum dots for advanced photonic and optoelectronic applications.

### 5.1 Batch-to-batch reproducibility and synthetic variability

Despite the rapid progress in the synthesis of MODs, batch-to-batch reproducibility remains a significant challenge that limits both fundamental studies and large-scale applications. Most MQDs are produced *via* top-down approaches, such as chemical etching, hydrothermal cutting, or solvothermal fragmentation of layered MXenes. These methods are inherently sensitive to synthesis parameters, including etchant concentration, reaction temperature, duration, solvent environment, and post-treatment conditions. Small variations in these parameters can lead to substantial differences in MQD size distribution, thickness, surface termination density, and defect concentration, resulting in inconsistent optical properties across batches.

In contrast to bottom-up semiconductor quantum dots, where nucleation and growth kinetics can be tightly controlled, MQD synthesis often lacks precise control over the fragmentation process.^71,76^ The coexistence of monolayer and few-layer MQDs, irregular lateral dimensions, and heterogeneous edge structures further exacerbates variability. Additionally, surface terminations such as –O, –OH, and –F are strongly influenced by the etching chemistry and washing procedures, leading to non-uniform surface states that directly affect photoluminescence behavior.

This reproducibility issue complicates meaningful comparison between studies, as reported optical properties often depend strongly on synthesis-specific conditions rather than intrinsic material characteristics. Without standardized synthetic protocols and characterization benchmarks, it remains difficult to establish reliable structure–property relationships, posing a major obstacle to both mechanistic understanding and technological translation of MQDs.

### 5.2 Quantum yield inconsistency and measurement challenges

Another critical limitation in MQD research is the large inconsistency in reported photoluminescence quantum yields (QYs), even for nominally similar material systems. Reported QY values for MQDs span a wide range, from below 1% to over 50%, reflecting substantial variability in surface chemistry, defect density, and post-synthetic modification. Unlike conventional semiconductor quantum dots, where high QYs are typically achieved through well-defined core–shell passivation strategies, MQD emission is dominated by surface and defect-related states that are highly sensitive to subtle chemical variations.

Surface functional groups, heteroatom dopants, and edge reconstruction can either enhance radiative recombination or introduce nonradiative decay pathways. As a result, MQDs synthesized under slightly different conditions may exhibit dramatically different emission efficiencies, even if their size distributions are comparable.^29,75^ Furthermore, excitation-dependent emission commonly observed in MQDs complicates QY determination, as measured values may depend on excitation wavelength and power density.

In addition to intrinsic material variability, inconsistencies also arise from differences in experimental protocols. Variations in reference standards, solvent environments, optical densities, and correction methods can significantly affect reported QY values. In some cases, insufficient consideration of scattering, reabsorption, or inner-filter effects may lead to overestimated efficiencies. These issues collectively hinder the establishment of reliable performance benchmarks for MQDs and make it challenging to assess genuine progress in luminescence optimization across different studies.

### 5.3 Environmental stability and toxicity considerations

Although MQDs are often regarded as a potentially safer alternative to heavy-metal-based semiconductor quantum dots, concerns regarding environmental stability and toxicity remain insufficiently addressed. MQDs contain transition metals such as Ti, V, or Nb, whose long-term chemical stability and potential ion release under physiological or environmental conditions are not yet fully understood. Surface oxidation, hydrolysis, or degradation may alter both optical properties and biocompatibility, particularly under prolonged light exposure or in aqueous and biological environments.

Residual etching agents, fluorine-containing surface terminations, and organic solvents used during synthesis and purification may further contribute to cytotoxicity or environmental risks if not adequately removed. Moreover, the small size and high surface reactivity of MQDs raise concerns regarding bioaccumulation, cellular uptake, and unintended interactions with biomolecules.^38–41^ Current toxicity assessments are often limited to short-term *in vitro* studies, providing an incomplete picture of long-term effects.

Environmental stability also poses challenges for device integration, as exposure to moisture, oxygen, or elevated temperatures can induce surface reconstruction and PL degradation. Without systematic evaluation of degradation pathways and metal leaching behavior, the safe deployment of MQDs in biomedical, wearable, or large-area optoelectronic applications remains uncertain. Addressing these stability and toxicity concerns is essential for establishing MQDs as truly sustainable and application-ready luminescent nanomaterials.

Table 4 outlines the primary scientific and technological challenges hindering the development of MQDs. These include poor batch reproducibility, inconsistent quantum yields, environmental sensitivity, and potential toxicity from transition metal cores or surface terminations. Addressing these issues requires standardized synthesis protocols, improved surface control, reliable quantification of luminescent efficiency, and systematic evaluation of long-term stability to enable scalable production and safe integration into advanced optoelectronic applications.

**Table 4:** Major challenges and limitations in the development and application of MQDs

Challenge	Primary cause	Key factors affected	Impact on photoluminescence	Current mitigation strategies
Batch-to-batch variability	Sensitive synthesis parameters	Size distribution, surface terminations	Inconsistent spectral properties	Standardized protocols, automation
Limited size control	Uncontrolled fragmentation	Quantum confinement regime	Irregular emission peaks	Optimizing etching and reaction kinetics
Low or inconsistent quantum yield	Surface and defect heterogeneity	Radiative *vs.* nonradiative rates	Strong QY fluctuations	Surface passivation, doping
Measurement inconsistency	Varied experimental protocols	Reference standards, solvents	Unreliable QY reporting	Unified optical measurement guidelines
Environmental instability	Oxidation, hydrolysis	Surface states, PL lifetimes	Degraded emission efficiency	Encapsulation, inert atmospheres
Potential toxicity	Metal ion release, residual etchants	Biocompatibility, cytotoxicity	Limited biomedical use	Post-synthesis purification
Scalability limitations	Complex etching chemistry	Reproducibility, uniformity	Low manufacturing throughput	Continuous-flow synthesis
Lack of long-term stability data	Poor understanding of degradation	Structural integrity	Unpredictable long-term behavior	Aging and leaching studies

## 6 Conclusions and future perspectives

MQDs have emerged as an important class of zero-dimensional nanomaterials that extend the functionality of two-dimensional MXenes into the quantum regime. Owing to strong quantum confinement, tunable surface chemistry, and abundant edge states, MQDs exhibit unique photoluminescence (PL) behaviors that can be engineered across a wide spectral range. In contrast to conventional semiconductor quantum dots, the emission properties of MQDs are governed by a complex interplay between the intrinsic electronic structure of the MXene core and extrinsic factors such as surface terminations, defects, and functional groups. This structural and chemical tunability has enabled MQDs to demonstrate promising performance in applications including optoelectronic devices, bioimaging, optical sensing, and advanced photonic systems.

Recent studies have shown that strategies such as heteroatom doping, controlled surface passivation, and hybrid material design can significantly enhance MQD optical properties. For instance, sulfur- and nitrogen-doped Ti_3_C_2_ MQDs exhibit excitation-independent multicolor emission and improved quantum yields, making them attractive for white light-emitting devices and bioimaging platforms.^24–29^ Similarly, controlled synthesis of Ti_2_C MQDs has enabled ultranarrow laser emission, while V_2_C MQDs have demonstrated white random lasing behavior based on nonlinear scattering processes. Advances in fluorine-free and low-temperature synthesis routes, such as those reported for Ti_2_N MQDs, also highlight the importance of environmentally benign preparation methods for achieving stable UV emission and minimizing non-radiative recombination processes. These developments collectively underline the strong relationship between synthesis conditions, structural characteristics, and photophysical behavior in MQDs.

Despite these advances, several critical challenges remain that must be addressed before MQDs can be widely implemented in practical technologies. One major issue is the difficulty of achieving consistently high quantum yields across the visible and ultraviolet regions while maintaining long-term photostability. Precise control over size distribution, thickness, and defect density during synthesis is still challenging, and variations in surface terminations can significantly influence emission characteristics and reproducibility.^81–83^ In addition, the fundamental mechanisms governing multicolor emission, excitation-dependent photoluminescence, and nonlinear optical responses remain incompletely understood. Addressing these questions will require the integration of advanced characterization techniques, such as time-resolved spectroscopy and ultrafast optical measurements, together with theoretical modeling approaches including time-dependent density functional theory to clarify exciton formation, recombination pathways, and charge transfer processes.

Looking forward, future research should focus on developing scalable and controllable synthesis strategies that enable precise engineering of MQD size, composition, and surface chemistry. Improved surface passivation and defect engineering strategies will be essential for enhancing photoluminescence efficiency and long-term stability. In parallel, the development of environmentally stable and low-toxicity MQDs will be particularly important for biomedical and wearable technologies. The integration of MQDs with other functional materials, including polymers, plasmonic nanostructures, and flexible substrates, may further expand their capabilities in light-emitting devices, optical sensors, and flexible photonic systems.

In addition to conventional optoelectronic applications, MQDs offer exciting opportunities in emerging areas of photonics.^84–87^ Their tunable emission and strong nonlinear optical responses make them promising candidates for ultrafast photonics, compact laser systems, and high-resolution optical devices. Furthermore, MQDs with excitation-independent full-color emission could enable next-generation display technologies, while UV-emitting MQDs may open new possibilities in phototherapy, sterilization, and environmental sensing. The potential integration of MQDs into quantum photonic architectures and light-harvesting systems also represents an intriguing direction for future investigation.

Overall, MQDs represent a rapidly evolving platform with significant potential in nanophotonics and optoelectronics. Continued progress will depend on a deeper mechanistic understanding of their photophysical properties, improved control over synthesis and surface chemistry, and effective integration into functional device architectures. Through coordinated advances in materials design, characterization, and device engineering, MQDs are expected to play an increasingly important role in the development of next-generation photonic and optoelectronic technologies.

## Conflicts of interest

The authors declare that they have no known competing financial interests or personal relationships that could have appeared to influence the work reported in this paper.

## Data Availability

This article is a review and does not include any new experimental data. All data discussed and analyzed are derived from previously published studies, which are appropriately cited in the manuscript.
